# Effect of strontium on transcription factors identified by transcriptome analyses of bovine ruminal epithelial cells

**DOI:** 10.1186/s12917-024-03929-9

**Published:** 2024-03-08

**Authors:** Panpan Tan, Yazhou Wang, Linshan Mei, Juan J. Loor, Chenxu Zhao, Yezi Kong, Fangyuan Zeng, Baoyu Zhao, Jianguo Wang

**Affiliations:** 1 College of Veterinary Medicine, Northwest A&F University, Yangling, 712100 Shaanxi China; 2 Department of Animal Sciences, Division of Nutritional Sciences, University of Illinois, Urbana, IL 61801 USA

**Keywords:** Strontium, Transcription factors, Cell differentiation, Lipid metabolism

## Abstract

**Background:**

Strontium (Sr) has similar physicochemical properties as calcium (Ca) and is often used to evaluate the absorption of this mineral. Because the major route of Ca absorption in the bovine occurs in the rumen, it is essential to understand whether Sr impacts the ruminal epithelial cells and to what extent.

**Results:**

In the present study, RNA sequencing and assembled transcriptome assembly were used to identify transcription factors (TFs), screening and bioinformatics analysis in bovine ruminal epithelial cells treated with Sr. A total of 1405 TFs were identified and classified into 64 families based on an alignment of conserved domains. A total of 174 differently expressed TFs (DE-TFs) were increased and 52 DE-TFs were decreased; the biological process-epithelial cell differentiation was inhibited according to the GSEA-GO analysis of TFs; The GO analysis of DE-TFs was enriched in the DNA binding. Protein-protein interaction network (PPI) found 12 hubs, including *SMAD4*, *SMAD2*, *SMAD3*, *SP1*, *GATA2*, *NR3C1*, *PPARG*, *FOXO1*, *MEF2A*, *NCOA2*, *LEF1*, and *ETS1*, which verified genes expression levels by real-time PCR.

**Conclusions:**

In this study, *SMAD2*, *PPARG*, *LEF1*, *ETS1*, *GATA2*, *MEF2A*, and *NCOA2* are potential candidates that could be targeted by Sr to mediate cell proliferation and differentiation, as well as lipid metabolism. Hence, these results enhance the comprehension of Sr in the regulation of transcription factors and provide new insight into the study of Sr biological function in ruminant animals.

**Supplementary Information:**

The online version contains supplementary material available at 10.1186/s12917-024-03929-9.

## Background

Strontium (Sr) is a Group II alkaline-earth metal with similar physicochemical properties to calcium (Ca). As a bone-seeking element, Sr has dual effects in regulating bone metabolism, on the one hand, Sr promotes bone formation by stimulating osteoblasts, and on the other hand, Sr suppresses bone resorption by inhibiting osteoclasts, resulting in increased bone deposition rates and bone mineral density [1, 2]. It has been widely used to treat osteoporosis in the form of Sr ranelate to promote osteogenic proliferation and differentiation via various pathways [3–6]. In primary bovine chondrocytes, Sr promotes proliferation and inhibits differentiation via the TGFβ/SMAD pathway [7]. In sheep and dairy cows, Sr concentration in the blood plasma can serve as an index of ruminal Ca absorption capacity under different states of Ca homeostasis [8, 9]. Our previous data uncovered some underlying targets of Sr-mediated Ca^2+^ metabolism regulation in bovine ruminal epithelial cells [10]. However, it is unknown what effect Sr has on transcription factors (TF) that regulate Ca^2+^ metabolism, proliferation, and differentiation.

By binding to enhancer and promoter regions in DNA sequences, TFs play a crucial role in the regulation of gene expression with key functions in numerous cellular processes, such as development and differentiation, stress responses, and response to external signals [11–14]. Research suggested that TFs including the krüppel-like transcriptional factors (KLFs) family, *NRF1*, *SMAD3*, *PPARG*, and *ATF4*, *SIRT4* play roles in the formation and development of bovine adipogenesis, glucose homeostasis, and the regulation of lipid metabolism [15–19]. The TF early growth response 1 (*EGR1*) promotes bovine skeletal muscle-derived satellite cell differentiation, and Sr has the ability to regulate the expression levels of TFs such as *SOX9*, which regulates osteogenic and adipocytic differentiation, articular cartilage degeneration, and subchondral bone remodeling [2, 20–22]. Sr can also inhibit TF such as *PPARG2*, rapidly reducing adipogenesis and thereby suppressing lipid droplet production [23, 24]. Additionally, by inhibiting the *NF-κB* activation, Sr is involved in the anti-inflammatory response and helps reduce the inflammatory cytokines [25, 26]. Whether Sr is able to affect TFs in ruminal epithelial cells is unknown.

The specific objective of the present study was to use RAN-seq-based transcriptome data to identify the effect of varying doses of Sr on TFs in bovine ruminal epithelial cells. Bioinformatics analysis, including protein-protein interaction networks, and then RT-PCR validation, were used to determine potential biological functions influenced by Sr and the affected TFs.

## Results

### Overview of RNA sequencing results

After removing short raw reads and quality inspection, the RNA sequence produced 53.1, 55.2, 55.6, 54.3, 48.5, and 49.5 million, respectively, clean reads from the six libraries. The GC content percentages were 55.36%, 55.59%, 55.81%, 55.61%, 54.63%, and 55.49%, respectively. The Q20 and Q30 values ranged from 96.90 to 97.22%, 91.56–92.17%, respectively. The reads mapped to the bovine genome all exceeded 96% (Table 1).

**Table 1:** Summary of of basic statistics for the rumen epithelial cell transcriptome sequencing data

Sample	Raw Datas	Clean Data (%)	Total Mapped (%)	Unique_Mapped(%)	Q20 (%)	Q30 (%)	GC Content (%)
Sr_0–1	53,338,342	53,161,530 (99.67%)	51,471,868 (97.26%)	49,878,179 (94.25%)	97.22%	92.17%	55.36%
Sr_0–2	55,476,744	55,289,308 (99.66%)	53,605,616 (97.38%)	51,902,730 (94.28%)	97.22%	92.15%	55.59%
Sr_0–3	55,825,190	55,631,872 (99.65%)	53,626,105 (96.82%)	51,928,516 (93.76%)	96.91%	91.59%	55.81%
Sr_20 − 1	54,576,396	54,394,994 (99.67%)	52,501,122 (96.90%)	50,976,998 (94.09%)	96.99%	91.74%	55.61%
Sr_20 − 2	48,728,294	48,581,650 (99.70%)	46,958,556 (97.08%)	45,603,478 (94.28%)	96.99%	91.63%	54.93%
Sr_20 − 3	49,708,604	49,544,270 (99.67%)	47,757,597 (96.80%)	46,339,132 (93.93%)	96.90%	91.56%	55.49%

### Identification and classification of TFs

**Fig. 1:**
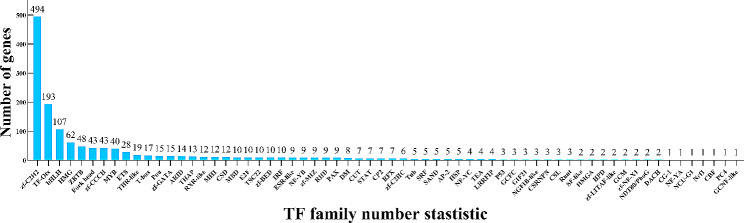
Summary of the TF family number stastistic. A total of 1405 TFs were identified and classified into 64 families via an alignment of conserved domains

**Fig. 2:**
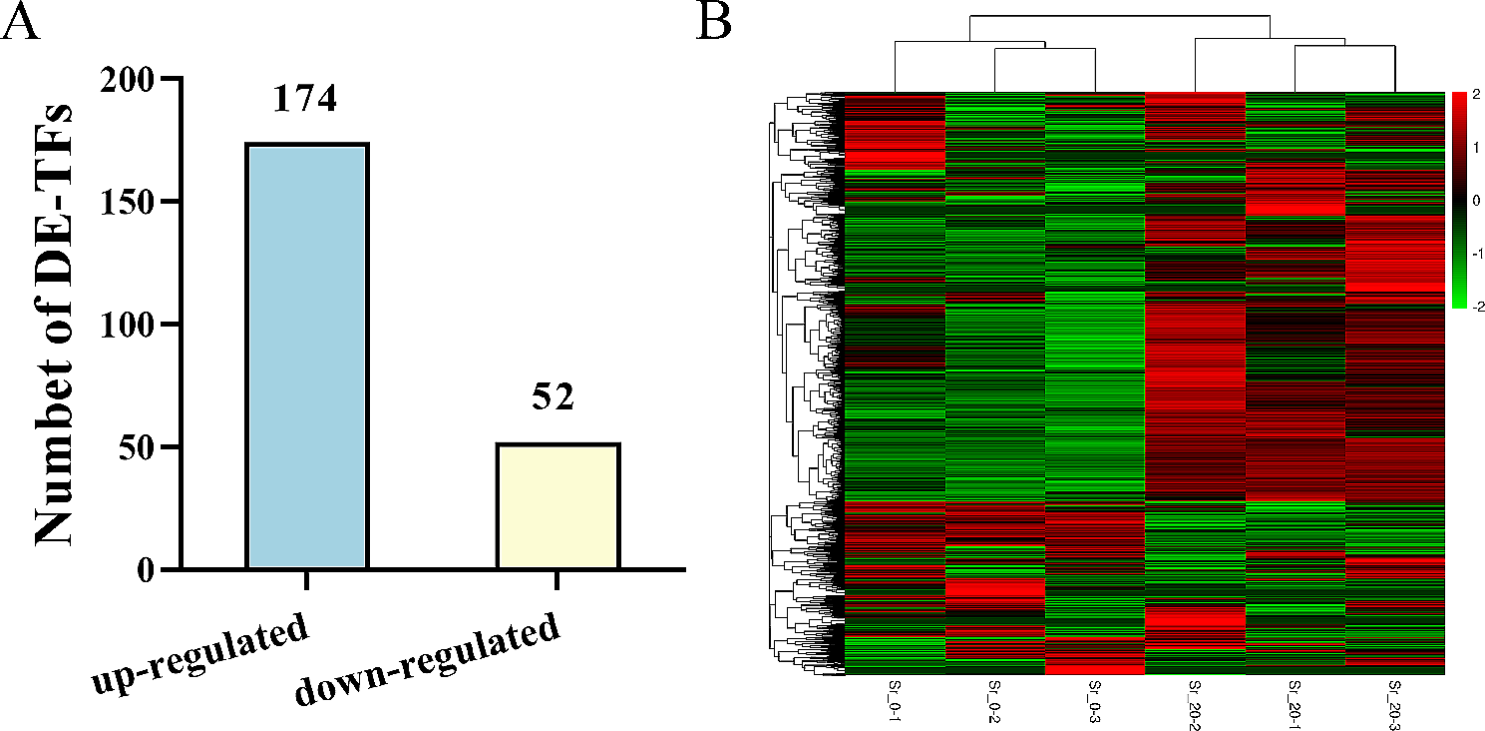
Summary of DE-TFs. **(A)** the number of up-regulated or down-regulated TFs, a total of 226 DE-TFs, specifically 174 DE-TFs showed up-regulated and 52 DE-TFs showed down-regulated; **(B)** Hierarchical clustering analysis of DE-TFs

As shown in Fig. 1, a total of 1405 TFs were identified and classified into 64 families via an alignment of conserved domains. The zf-C2H2 family of TFs contains the largest number of genes, followed by TF-Otx and bHLH. Further analysis revealed that the number of DE-TFs was 226, specially 174 DE-TFs showed up-regulated and 52 DE-TFs showed down-regulated (Fig. 2A); the DE-TFs of clustering analysis showed the expression patterns of most TFs (Fig. 2B).

### GSEA analysis of TFs

According to the GSEA-GO results, TFs were mainly enriched in biological processes such as inner ear morphogenesis, inner ear development, membrane part, integral component of membrane, epithelial cell differentiation, and regulation of muscle cell differentiation. Among these biological processes, regulation of muscle cell differentiation was activated (*P-*value < 0.05), whereas others were inhibited.

**Fig. 3:**
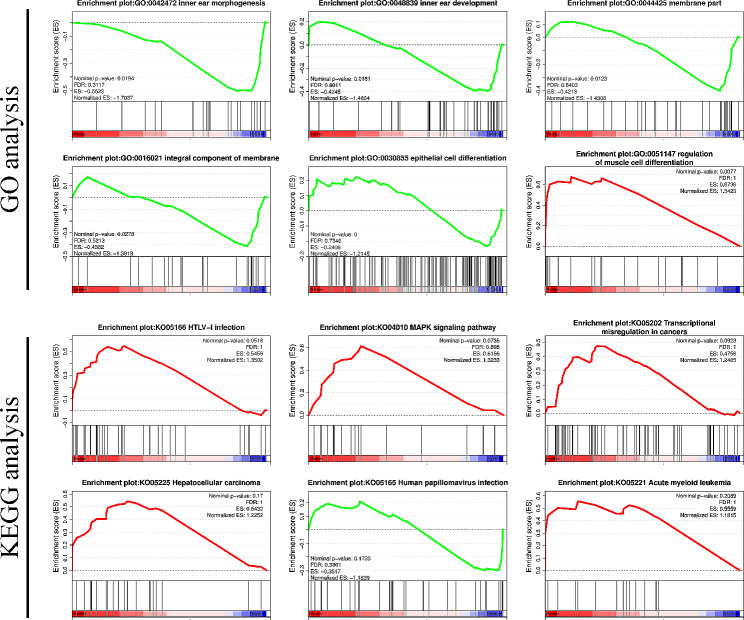
GSEA analysis results. The GSEA-GO results showed that among these biological processes, regulation of muscle cell differentiation was activated (*P*-value < 0.05), whereas others were inhibited. the GSEA-KEGG results showed that a total of 28 pathways were enriched (*P*-value > 0.05)

According to the GSEA-KEGG results, a total of 28 pathways were enriched (*P-*value > 0.05) after Sr treatment, including HTLV-I infection, MAPK signaling pathway, transcriptional misregulation in cancers, hepatocellular carcinoma, and human papillomavirus infection among others (Fig. 3).

### Gene ontology enrichment analysis of DE-TFs

**Fig. 4:**
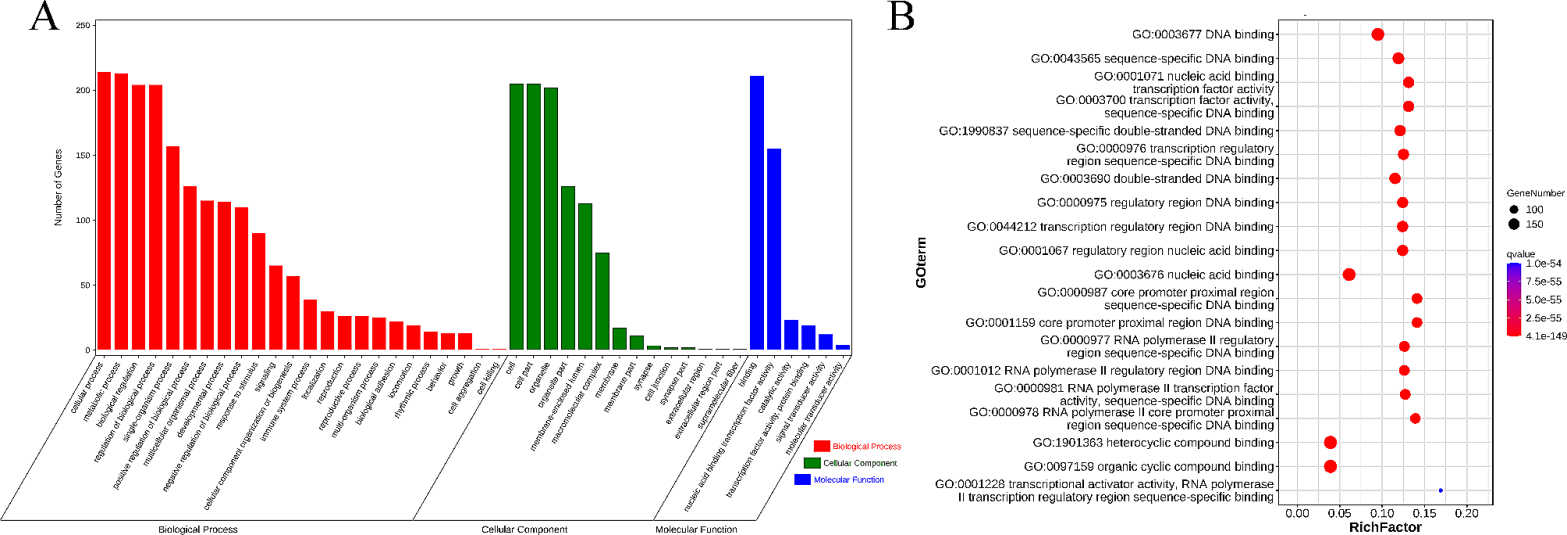
GO enrichment of DE-TFs. **(A)** The barplot of GO enrichment, mainly enriched in biological process; **(B)** The bubble chart of GO enrichment, the most significantly enriched terms involved in DNA binding, sequence-specific DNA binding, and nucleic acid binding transcription factor activity among others

GO enrichment analysis of DE-TFs revealed 772 GO terms (*q-*value < 0.05), including 86 terms in molecular function, 42 cellular components, and 644 in biological processes (Fig. 4A). The most significantly enriched terms involved in DNA binding, sequence-specific DNA binding, nucleic acid binding transcription factor activity, transcription factor activity-sequence-specific DNA binding, and sequence-specific double-stranded DNA binding among others (Fig. 4B).

### KEGG pathway analysis of DE-TFs

**Fig. 5:**
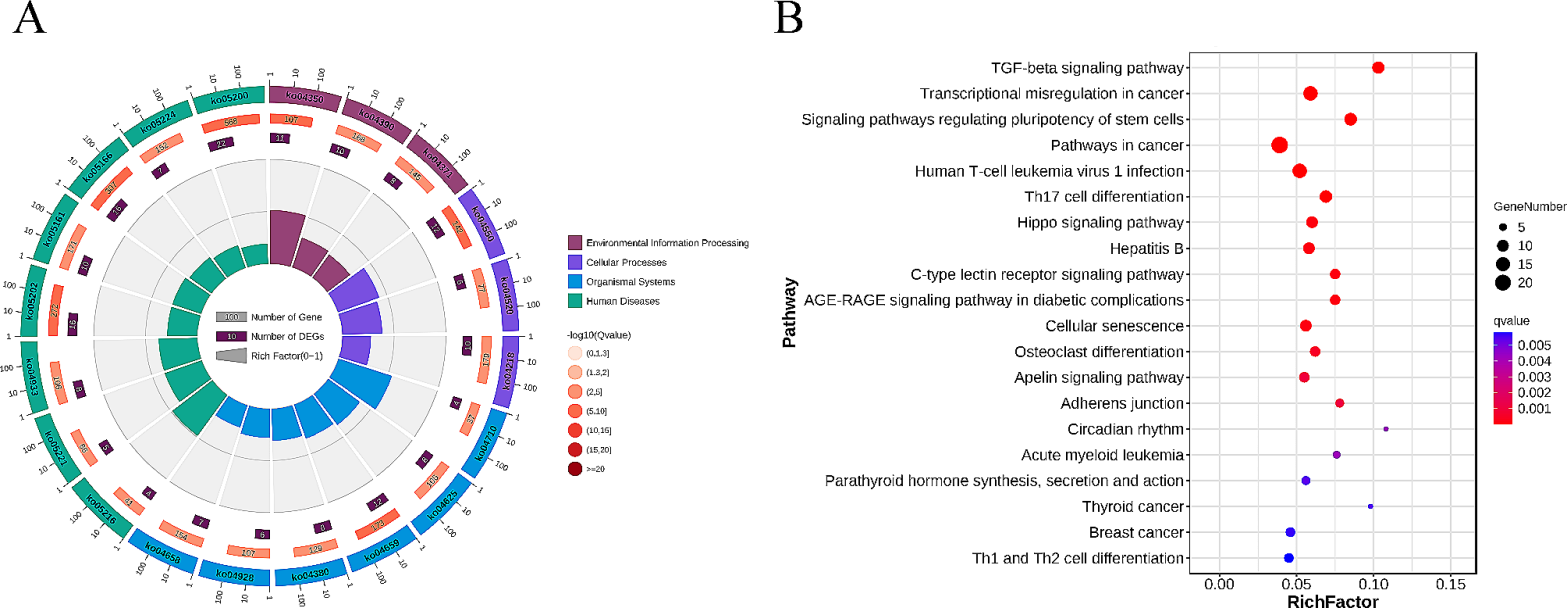
KEGG pathway enrichment of DE-TFs. **(A)** The circos plot of KEGG pathway enrichment, mainly enriched in human diseases, and organismal systems; **(B)** The bubble chart of KEGG pathway enrichment, 34 of which were significantly enriched (*P*-value < 0.05) in the TGF-β signaling pathway, transcriptional misregulation in cancer among others

DE-TFs were enriched in 135 pathways, a total of 34 of which were significantly enriched according to a *q-*value is less than 0.05. The top 20 pathways included the TGF-β signaling pathway, transcriptional misregulation in cancer, signaling pathways regulating pluripotency of stem cells, pathways in cancer, human T-cell leukemia virus 1 infection, and Th17 cell differentiation, among others (Fig. 5).

### PPI network construction

**Fig. 6:**
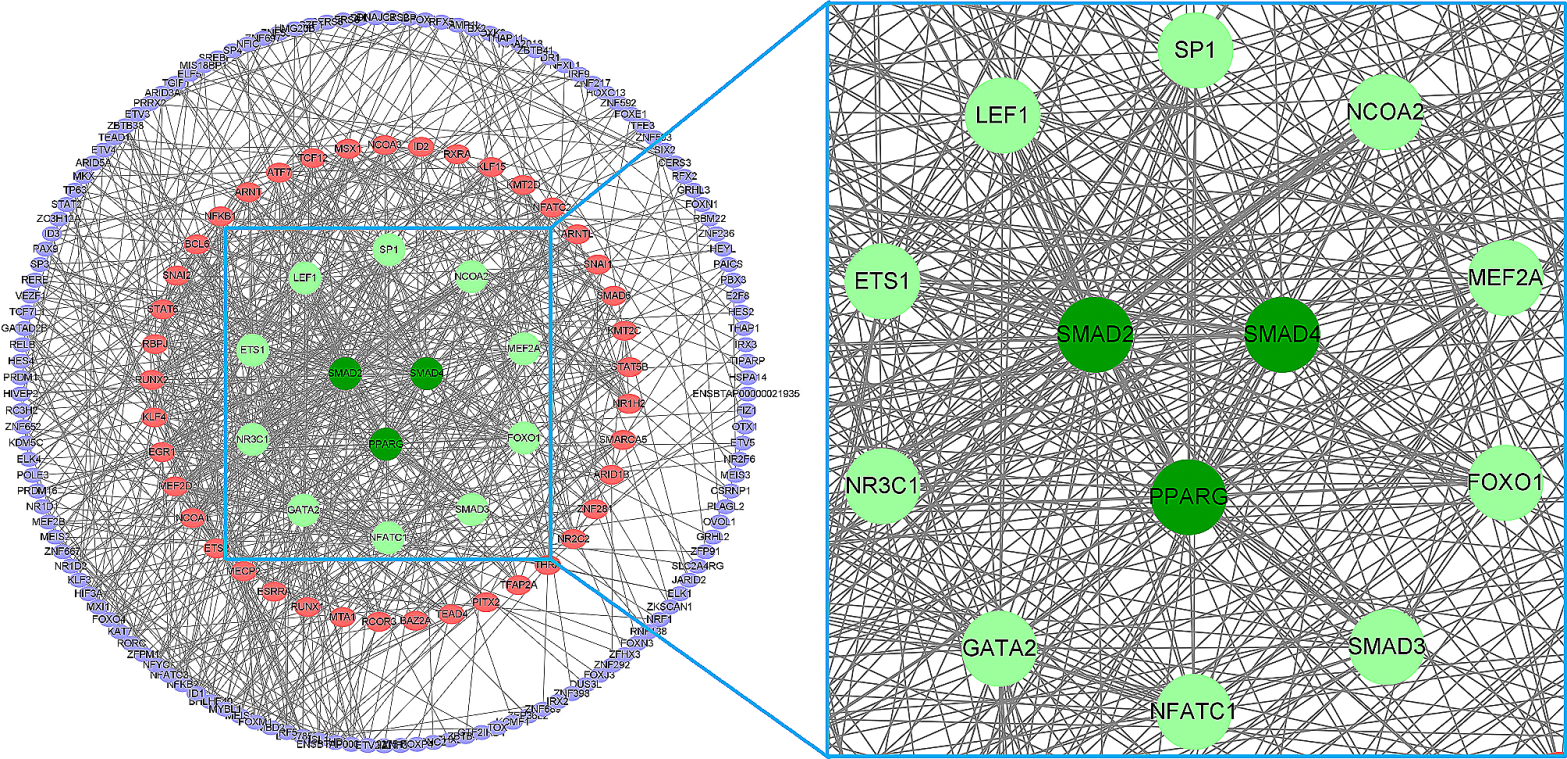
Screen of hub DE-TFs. PPI analysis showed that the DE-TFs encompassed a total of 49 core targets, with a total of 12 hub DE-TFs were detected, including *PPARG*, *SMAD4*, *SMAD2*, *SMAD3*, *FOXO1*, *MEF2A*, *NCOA2*, *SP1*, *LEF1*, *ETS1*, *NR3C1*, and *GATA2*

**Fig. 7:**
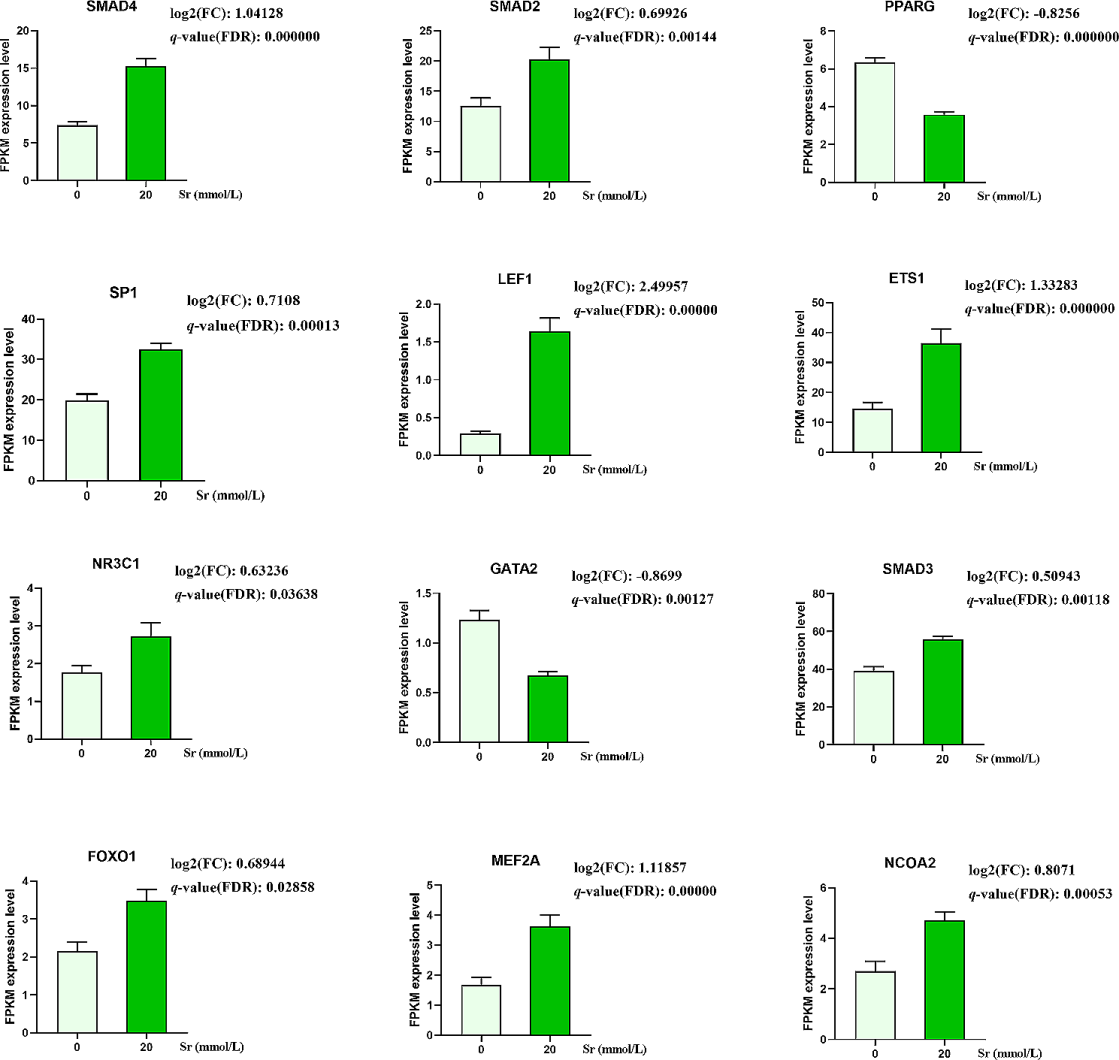
The hub DE-TFs expression levels in RNA-seq. The expression levels of *SMAD4*, *SMAD2*, *SP1*, *LEF1*, *ETS1*, *NR3C1*, *SMAD3*, *FOXO1*, *MEF2A* and *NCOA2* were up-regulated (*q*-value < 0.01 and *q*-value < 0.05), while the expression levels of *PPARG* and *GATA2* were down-regulated (*q*-value < 0.01)

Based on “betweenness”, “closeness”, and “degree”, PPI analysis via STRING and Cytoscape software indicated that the DE-TFs encompassed a total of 49 core targets (Fig. 6). A total of 12 hub DE-TFs were detected, among which the expression levels of *SMAD4*, *SMAD2*, *SP1*, *LEF1*, *ETS1*, *NR3C1*, *SMAD3*, *FOXO1*, *MEF2A*, and *NCOA2* were up-regulated, while the expression levels of *PPARG* and *GATA2* were down-regulated (Fig. 7).

### Verification of DE-TFs by RT-PCR

**Fig. 8:**
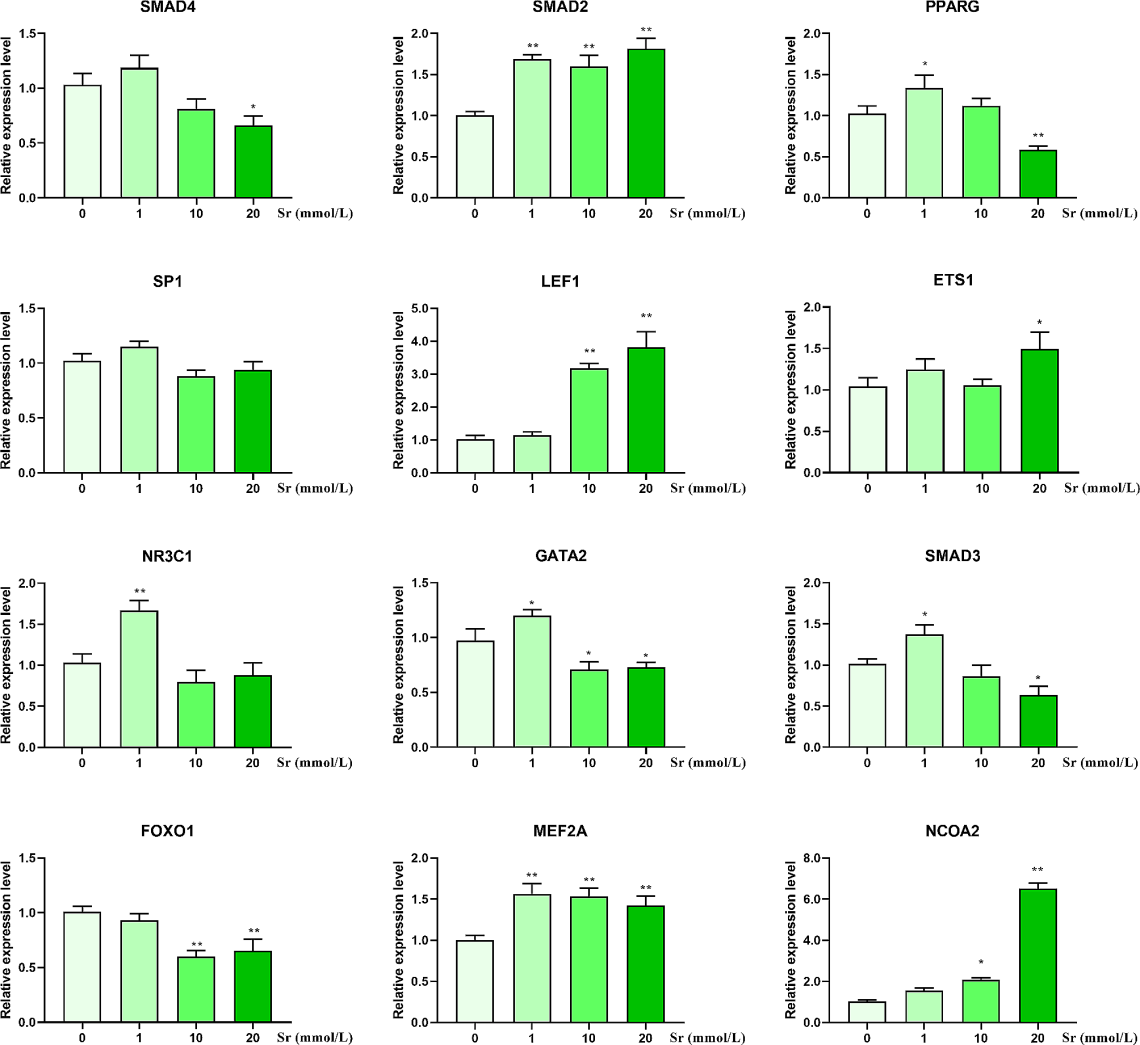
mRNA expression analysis of 12 DE-TFs genes at different doses of Sr. Data are presented as mean ± SEM, *P < 0.05, **P < 0.01 as compared with the 0 mmol/L Sr group

The results of RT‑PCR for 12 selected TFs are in Fig. 8. The *SMAD2* and *MEF2A* expression levels were up-regulated after treatment with Sr for 24 h. The expression of *LEF1* and *NCOA2* were up-regulated in response to 10 and 20 mmol/L Sr group. The *ETS1* was up-regulated in the 20 mmol/L Sr group. The expression levels of *PPARG*, *NR3C1*, *GATA2*, and *SMAD3* were up-regulated in the 1 mmol/L group and down-regulated in the 20 mmol/L group. The *FOXO1* expression level was down-regulated in the 10 and 20 mmol/L groups, the *SMAD4* expression level was down-regulated in the 20 mmol/L group, while the expression level of *SP1* was not affected by Sr treatment.

RT-PCR and RNA-seq analysis of *GATA2*, *ETS1*, *MEF2A*, *SMAD2*, *PPARG*, *LEF1*, and *NCOA2* in 20 mmol/L groups show a consistent expression changes, while the expression changes of *SMAD4*, *SMAD3*, and *FOXO1* detected by RT-PCR and RNA-seq display an opposite trends.

## Discussion

Transcription factors recognize specific DNA sequences to control chromatin and transcription, thus, play an important role in a wide variety of processes, including the regulation of gene expression, cellular function, and environmental responses [27, 28]. The AnimalTFDB v4.0 website contains information for 1445 TFs and 939 TF Cofactors for Bos taurus. From the sequencing results, we identified 1405 TFs classified into 64 families, among which Zf-C2H2, TF-Otx, and bHLH were dominant after Sr treatment. GO enrichment analysis of 226 DE-TFs revealed that “binding” was the highest GO category with DNA binding having 192 transcription factors, sequence-specific DNA binding having 165 transcription factors, and nucleic acid binding having 155 transcription factors. Such associations likely reflect the fact that all transcriptional events in living organisms require the binding of polyprotein complexes to DNA [29].

The KEGG enrichment analysis revealed that DE-TFs were enriched in pathways such as TGF-β signaling pathway, transcriptional misregulation in cancer, signaling pathways regulating pluripotency of stem cells, pathways in cancer, human T-cell leukemia virus 1 infection, and Th17 cell differentiation among others. TFs are involved in cell progression, such as cell differentiation, cell proliferation, response to external signals, among others, through regulating the expression of regulatory genes and all other genes [11–13]. Thus, it was important to explore hub TFs in response to treatment with Sr. The PPI analysis of DE-TFs after treatment with Sr revealed 12 hub-TFs: *SMAD4*, *SMAD2*, *SMAD3*, *SP1*, *GATA2*, *NR3C1*, *PPARG*, *FOXO1*, *MEF2A*, *NCOA2*, *LEF1*, and *ETS1*.

The TGF-β superfamily signaling regulates several cellular processes including cell differentiation, proliferation, apoptosis, inflammation, and fibrosis [30, 31]. SMAD proteins are the central players in the canonical TGF-β signaling pathway in which signaling triggers the phosphorylation of the receptor-activated SMADs (R-SMADs), *SMAD2* and *SMAD3* [32]. The activated R-SMADs bind the common mediator SMAD (co-SMAD), *SMAD4*, and the resulting complexes relocate into the nucleus [33–35]. For instance, a previous study reported that Sr increased the *SMAD2/3* and TGF-β protein expression levels and regulated the TGF-β/SMAD signaling pathway to promote osteogenic differentiation [36]. Similarly, our previous study observed that Sr regulated primary chondrocyte proliferation and differentiation by directing TGFβ1 signaling toward *SMAD3* phosphorylation [7].

Through binding to *SMAD4*, *GATA2* acts as a negative regulator of the TGF-β signaling pathway, and its overexpression decreases the DNA binding activity of *SMAD4* [37]. *ETS1* plays a role in osteoblast differentiation and bone development and is a downstream signaling effector for several *TGFβ1* responsive genes [38, 39]. Overexpression of *ETS1* prevented the *TGF-β*-induced reduction in DNA-binding activity [40]. Myocyte enhancer factor 2 A (*MEF2A*) inhibition abated activation of the TGF-β/SMAD signaling pathways [41]. The overexpression of specificity protein 1 (*SP1*) increased *TGF-β1* expression levels and activated the TGF-β1/SMAD2 signaling pathway, while the downregulation of *SP1* inhibited osteoblast differentiation [42, 43].

In the present study, the expression levels of *SMAD2*, *ETS1*, and *MEF2A* were significantly increased (*P* < 0.01 and *P* < 0.05) and the *GATA2* was significantly decreased (*P* < 0.05), which was consistent in RT-PCR and RNA-seq. However, the decreased expression levels *SMAD4* and *SMAD3* (*P* < 0.05) and the unchanged *SP1* as shown in RT-PCR were opposite to RNA-seq likely due to the interference of duplication in the sequencing results. Based on the GSEA analysis of TFs, KEGG enrichment analysis of DE-TFs and RT-PCR analysis, the altered expression levels of *SMAD2*, *ETS1*, *GATA2*, and *MEF2A* indicated that Sr may activate the TGF-β signaling pathway to regulate the differentiation of ruminal epithelial cells. The precise molecular mechanisms require further investigation.

The ruminal epithelium is the main site for ketogenesis from acetate and butyrate during the fed state, while in non-ruminants and ruminants ketogenesis occurs mainly in the liver during the post-absorptive or starvation states [44]. Both *PPARG* and *FOXO1* are regulatory proteins that play a crucial role in lipid metabolism [45–47]. *FOXO1* binds directly to the promoter of *PPARG* resulting in the suppression of its transcription, thereby decreasing adipogenesis [45, 48]. The overexpression of *FOXO1* in liver contributes to an increase in the synthesis of triglyceride (TGs) and a decrease in the oxidation of fatty acids, exacerbating hepatic steatosis [49]. *PPARG* serves as the main regulator of triacylglycerol synthesis and secretion, and its overexpression indeed induces lipid accumulation [50]. *NCOA2* (also referred to as *TIF2*), an activator of *PPARG*, is a master TF of adipogenesis and its knockdown can decrease *PPARG* activity thereby reducing fat accumulation in white adipose tissue [51–53]. Glucocorticoid receptor (*NR3C1*), a glucocorticoid receptor, is a modulator of hormonal regulation of immunity and lipid metabolism, and glucocorticoids up-regulate *FOXO1* expression by binding to *NR3C1* [54, 55]. Lymphocyte enhancer factor-1 (*LEF1*) is a member of the LEF1/T-cell factor (TCF) family, and it could interact with *β-catenin* to regulate adipogenesis and negatively regulate lipid deposition [56, 57].

In this study, the decreased expression of *PPARG* (*P* < 0.01) and the increased expression of *NCOA2* and *LEF1* expression levels (*P* < 0.01), were consistent in RT-PCR and RNA-seq. However, the *FOXO1* expression levels were decreased (*P* < 0.01) and the *NR3C1* expression levels were not significantly changed in the results of RT-PCR were opposite to RNA-seq for reasons that cannot be explained with the available data. The altered expression of *PPARG* and* LEF1* indicated that Sr plays an important role in lipid metabolism in ruminal epithelial cells.

## Conclusions

A large number of TFs were identified by RNA-seq in response to incubation with Sr, suggesting they may be involved in different regulatory pathways in ruminal epithelial cells. Seven potential targets for Sr-mediated cell proliferation and differentiation, and lipid metabolism regulation were identified: *SMAD2*, *PPARG*, *LEF1*, *ETS1*, *GATA2*, *MEF2A*, and *NCOA2*. Understanding the underlying molecular mechanisms of these TFs requires further studies. Overall, the results enhanced the knowledge regarding the impacts of Sr on transcriptional regulation in ruminal epithelial cells. Whether Sr could be used in practice to alter molecular mechanisms in vivo will have to be addressed in future research.

## Materials and methods

### Primary bovine rumen epithelial cells isolation, culture and treatment

The primary bovine ruminal epithelial cells were isolated as previously described [10, 58]. In brief, the newborn Holstein male calves (*n* = 3, 38.0 ± 2.8 kg body weight) were euthanized with veterinarian pentobarbital sodium at 100 mg/kg intravenous. The rumen tissue was collected and washed several times until no visible rumen contents remained. Then, the ruminal epithelium was bluntly dissected and washed with PBS buffer containing penicillin, streptomycin, and amphotericin B, gentamicin. The ruminal epithelium was aseptically cut into small pieces (3–4 cm^2^) and trypsinized via serial incubations with trypsin-EDTA solution. The subsequent 4 fractions of supernatant were each strained through 50-mesh and washed with PBS buffer. Then, the ruminal epithelium cell pellet was resuspended with DMEM-low-glucose medium and strained through 200-mesh. Lastlly, the cell density was adjusted to 1 × 10^6^ cells/mL, and seeded into six-well cell culture plates (2 mL per well) followed by incubation at 37 °C in 5% CO_2_ in a humidified incubator. The medium was changed every 2 days.

According to the IC50, cell cycle, and Ca^2+^ levels in our previous study, primary bovine ruminal epithelium cells were cultured up to 90% confluency, the original culture medium was discarded, and the cells were washed with PBS buffer. The fresh medium containing different doses of Sr (0, 1, 10, and 20 mmol/L) was added to the corresponding groups, and the cells were cultured in a 5% CO_2_ atmosphere at 37 °C for 24 h (three replicates for each group). Then the cells were washed twice with PBS buffer, collected by scraping with a cell scraper, and stored at -80℃ to be used for subsequent experiments.

### RNA isolations, libraries construction and sequencing

Total RNA from the experimental cells was extracted using TRIzol™ Reagent (Invitrogen, Carlsbad, CA, USA) according to the recommendations. After evaluation of the RNA quality assessed by Agilent 2100 Bioanalyzer (Agilent Technologies, Palo Alto, CA, USA) and checked using RNase free agarose gel electrophoresis, mRNA was enriched by Oligo(dT) beads. Then the enriched mRNA was fragmented into short fragments and reversed transcribed into cDNA by using NEBNext Ultra RNA Library Prep Kit for Illumina (NEB#7530, New England Biolabs, Ipswich, MA, USA). After synthesis of the second strand cDNA, fragments were end repaired and ligated to Illumina sequencing adapters. The ligation reaction was purified with the AMPure XP Beads (1.0X). Ligation products cDNA were size-selected by agarose gel electrophoresis and amplified by PCR, then the PCR products were purified with AMPure XP beads (1.0X), and the cDNA library was obtained. The resulting cDNA library was assessed the quality testing by agarose gel electrophoresis, NanoPhotometer spectrophotometer (Thermo Fisher Scientific, Massachusetts, USA), Qubit2.0 Fluorometer (Thermo Fisher Scientific, Massachusetts, USA), and Agilent 2100 bioanalyzer (Agilent Technologies, Palo Alto, CA, USA), and then sequenced using Illumina Novaseq6000 by Gene Denovo Biotechnology Co.(Guangzhou, China). The RNA-seq datasets were deposited in the NCBI Sequence Read Archive (SRA) with accession number SUB12106664.

RNA differential expression analysis was performed via DESeq2 software between two different groups. The genes with the parameter of false discovery rate (FDR) below 0.05 were considered differentially expressed gene (DEG).

### Identification of TFs

Unigenes that were annotated as transcription factor genes, for instance, bZIP, bHLH, and MYB, were then selected for further analysis. The differentially expressed transcription factor genes and identified temporal expression profiles were evaluated by the Short Time-series Expression Miner V1.3.13 (STEM) program.

### Functional GO and KEEG analysis

The functional analysis (GO and KEGG pathway) of DE-TFs was performed using the OmicShare tools at www.omicshare.com/tools. *q*-value is the *P*-value after multiple verification, and the GO function or KEGG pathway is considered to be significantly enriched if the *q*-value is less than 0.05.

A PPI was generated using STRING (https://cn.string-db.org/) and Cytoscape software (version 3.8.2) diagram to present the core and the biological interaction of hub genes.

### Gene set enrichment analysis (GSEA)

GSEA analysis was performed by using software GSEA and MSigDB to identify whether a set of genes in specific GO terms/KEGG pathways shows significant differences in control and Sr 20 mmol/L groups (www.omicshare.com/tools).

### Quantitative real-time polymerase chain reaction

After the total RNA was extraction, the cDNA was produced by reverse-transcribing the isolated total RNA using the FastKing RT Kit With gDNase (TIANGEN, Beijing). RT-PCR was performed in triplicate by using SYBR Green RT-PCR Master Mix (Vazyme, Nanjing). Primers were designed from span introns using NCBI primer design tool. The detailed primer information were shown in supplementary materials Table S1. *GAPDH* and *β-actin* were used as dual endogenous control. Relative quantification was done using the 2^−ΔΔCT^ method.

### Statistical analysis

RNA-seq analysis: the raw reads were filtrated by fastp (version 0.18.0) and base quality analysis to clean data, the clean data were blasted to the ribosomal RNA for unmapped reads; then the unmapped reads were aligned with the reference genome mapped to the *Bos taurus* reference genome (Ensembl_release104) using HISAT2. 2.4 software (http://www.ccb.jhu.edu/software/hisat/ accessed on 15 August 2021). The unmapped reads of each sample were assembled using StringTie v1.3.1 (https://ccb.jhu.edu/software/stringtie/ accessed on 15 August 2021) in a reference-based approach. A fragment per kilobase of transcript per million mapped reads (FPKM) value was calculated to quantify its expression abundance and variations, using RSEM software. RNA differential expression analysis was performed with DESeq2 (version 1.40. 1) software between the 0 mmol/L (Sr-0) and 20 mmol/L (Sr-20) Sr treatment groups.

All results are presented as mean ± standard deviation (SEM). GraphPad Prism 5.01 software and Statistical Package for the Social Sciences (SPSS 17.0) packages were used for statistical analyses. The *P* values were determined using one-way ANOVA among the various groups, and statistically significant was determined at *P* < 0.05.

### Electronic supplementary material

Below is the link to the electronic supplementary material.

**Supplementary Material 1: Table S1** Sequences used for RT-PCR

## Data Availability

The raw Illumina sequencing data were archived in the NCBI Sequence Read Archive (NCBI SRA) under accession number SUB12106664.

## Abbreviations

DEG: Differentially expressed gene
DE-TFs: Differentially expressed TFs
RT-PCR: Real-time polymerase chain reaction
*SMAD4*: SMAD family member 4
*SMAD2*: SMAD family member 2
*SMAD3*: SMAD family member 3
*SP1*: Specificity protein 1
*GATA2*: GATA binding protein 2
*NR3C1*: Glucocorticoid receptor
*PPARG*: Peroxisome proliferator-activated receptor-gamma
*FOXO1*: Forkhead box-O1
*MEF2A*: Myocyte enhancer factor 2 A
*NCOA2*: Nuclear receptor coactivator 2
*LEF1*: Lymphocyte enhancer factor-1
*ETS1*: ETS proto-onco 1
TGs: Triglyceride
