# Supplementary material for: Effect of strontium on transcription factors identified by transcriptome analyses of bovine ruminal epithelial cells
Source: BMC Vet Res. 2024 Mar 8;20:88. doi: 10.1186/s12917-024-03929-9 (PMC10921748; doi:10.1186/s12917-024-03929-9)
Supplement: Supplementary file 1 — Supplementary Material 1: Table S1 Sequences used for RT-PCR [file 12917_2024_3929_MOESM1_ESM.docx]

Supplementary materials

Table S1 Sequences used for RT-PCR

| **Gene symbol** | **Accession No.** | **Product size** | **Primer sequence (5’ → 3’)** |
| --- | --- | --- | --- |
| SMAD4 | NM_001076209.1 | 286 bp | F: TGTGGCTTCCACAAGTCAGC  R: AGGCTGGAATGCAAGCTCAT |
| SMAD2 | NM_001046218.1 | 267 bp | F: TGGGGAAGTTTTTGCCGAGT  R: ACCGTCTGCCTTCGGTATTC |
| PPARG | NM_181024.2 | 156 bp | F: AGACGACAGACAAATCACCGTT  R: CTTCCACGGAGCGAAACTGA |
| SP1 | NM_001078027.1 | 190 bp | F: TGCTACCATGAGCGACCAAG  R: CAAAGGGGATGGCTGGGATT |
| LEF1 | NM_001192856.1 | 252 bp | F: CGGAGCGGAGATTACACAGT  R: TTGTCTGGCCACCTCGTGTC |
| ETS1 | NM_001099106.2 | 270 bp | F: AACAGCAAACTCGCTCTAGCC  R: GTCCACTGCCGGGGATCTTT |
| NR3C1 | NM_001206634.1 | 279 bp | F: GGCCAGATGTACCACTACGA  R: TAGGCAGAGTTTGGGAGGTG |
| GATA2 | NM_001192114.3 | 113 bp | F: TTGCGCAAACTGTCAGACGA  R: GCGGCCTGTTCACGTTGTG |
| SMAD3 | NM_001205805.1 | 164 bp | F: GCAGAACGTCAACACCAAGT  R: CGAACTCACACAGCTCCATG |
| FOXO1 | XM_025000053.1 | 167 bp | F: CAAGCGAGCAAGCAGGCTAC  R: GCTGCCAAGTCTGACGAAAG |
| MEF2A | NM_001083638.2 | 255 bp | F: CGGAATCATAAAATCGCACCCG  R: AGTGCTCAACATCCCACCTG |
| NCOA2 | XM_015474472.2 | 202 bp | F: GGATTGTTGGAAGCACTTGATTT  R: TTCTCGGTGCTCCTTTTGGG |
| GAPDH | NM_001034034 | 117 bp | F: CCTGCCAAGTATGATGAGAT  R: AGTGTCGCTGTTGAAGTC |
| β-actin | NM_173979.3 | 159 bp | F: CAAGTACCCCATTGAGCACG  R: GTCATCTTCTCACGGTTGGC |
